# Selenium supplementation in patients undergoing hematopoietic stem cell transplantation: effects on pro-inflammatory cytokines levels

**DOI:** 10.1186/2008-2231-22-51

**Published:** 2014-06-17

**Authors:** Nesa Daeian, Mania Radfar, Zahra Jahangard-Rafsanjani, Molouk Hadjibabaie, Ardeshir Ghavamzadeh

**Affiliations:** 1 Department of Clinical Pharmacy, Faculty of Pharmacy, Tehran University of Medical Sciences, Enqelab SQ, 16 Azar AV, PO Box: 14155/6451, Tehran, Iran; 2 Research Center for Rational Use of Drugs, Tehran University of Medical Sciences, Tehran, Iran; 3 Hematology-Oncology and Stem cell Research Center, Shariati Hospital, Tehran University of Medical Sciences, Tehran, Iran

**Keywords:** Selenium, TNF-α, IL-6, Hematopoietic stem cell transplantation, Oral mucositis

## Abstract

**Background:**

Pro-inflammatory cytokines including tumor necrosis factor alpha (TNF-α), interleukin-1β (IL-1β) and interleukin-6 (IL-6) play an important role in the development of hematopoietic stem cell transplantation (HSCT) complications. We explored the effect of Selenium as an antioxidant and anti-inflammatory agent on pro-inflammatory cytokines levels in HSCT candidates.

**Findings:**

Plasma concentrations of TNF-α, IL-1β and IL-6 were measured in 74 patients from a double-blind, randomized, placebo-controlled study. In both groups, there were 37 patients with median age of 32 years. Patients received oral Se tablets (200 mcg) or placebo twice daily beginning from the first day of high dose chemotherapy (HDC) through 14 days after HSCT. Cytokine levels were determined before starting HDC (prior to first dose of Se), 7 and 14 days after HSCT. Plasma levels of TNF-α were not significantly different between Se and control group (*P* = 0.13). IL-1 levels were similar between two groups (*P* = 0.88). No significant differences were detected in IL-6 levels between Se and control group (*P* = 0.96).

**Conclusion:**

Selenium had no effect on pro-inflammatory cytokines levels in patients undergoing HSCT. It is likely that earlier initiation and/or larger doses of Se are required to affect inflammatory cytokines significantly.

## Findings

Hematopoietic stem cell transplantation (HSCT) is one of the most effective treatments for hematologic disorders. It is applicable to many patients due to substantial advances in the understanding of transplant immunology as well as better supportive care [1]. Despite these improvements, transplant-related complications still remain as major limitations of this curative modality. The serious and potentially life-threatening complications include mucositis, hepatic veno-occlusive disease, graft-versus-host disease (GVHD) and infections [2]. It has been demonstrated that pro-inflammatory cytokines including tumor necrosis factor alpha (TNF-α), interleukin-1β (IL-1β) and interleukin-6 (IL-6) play an important role in the development of these complications [3-5].

High dose chemotherapy (HDC) prior to HSCT is one of the main triggers of pro-inflammatory cytokines release. Oxidative stress and reactive oxygen species (ROS), produced by the chemotherapeutic agents, are activators of a number of transcription factors, such as nuclear factor-κB. This factor is responsible for up-regulating the genes which results in the production of pro-inflammatory cytokines including TNF-α, IL-1β, and IL-6 [4,6]. Hence, therapeutic agents which reduce oxidative stress or pro-inflammatory cytokines levels could be suggested to confront complications of HSCT. Previous studies have confirmed significant positive effects of administrating such agents including amifostine and TNF-α inhibitors in prevention or amelioration of transplant-related complications like oral mucositis (OM) and acute GVHD [7,8]. However based on our knowledge no similar study was conducted to evaluate the effects of selenium (Se) supplementation in HSCT setting.

Selenium, in the form of selenoproteines in particular glutathione peroxidase (Glu.Px), is an essential component of human cellular antioxidant defense system. It also has anti-inflammatory effects by scavenging free radicals [9].

We conducted a controlled study that indicated the efficacy of Se supplementation in reducing the incidence of severe oral mucositis (WHO grade 3–4) in HSCT setting [10]. In addition, significant improvements in serum Se concentration and plasma Glu.Px activity in Se group were revealed. In the present article, we report the effect of Se supplementation on plasma pro-inflammatory cytokine (TNF-α, IL-1β and IL-6) levels in order to illustrate possible underlying mechanisms of observed clinical outcomes.

The main study was a double-blind, randomized, placebo-controlled clinical trial conducted from June 2011 to July 2012 in the Hematology–Oncology and Stem Cell Transplantation Research Center (Dr. Shariati Hospital), Tehran University of Medical Sciences, Tehran, Iran. The study was registered in clinicaltrial.org (ID: NCT01432873) and was approved by the institutional ethics committee (ID: 900513) and written informed consent was obtained from all patients before study entry.

Adult patients with diagnosis of AML or ALL, candidates for allogeneic HSCT, were eligible for inclusion in the study. The other criteria were Karnofsky performance status >70% as well as adequate cardiac, pulmonary, renal and hepatic function according to the institutional protocol. Eligible patients were randomly assigned to receive either Se tablets (Webber Naturals, Coquitlam, BC, Canada, 200 mcg) or placebo using balanced blocked randomization. The researchers, patients and clinical staff were blinded to the randomization.

Patients received two tablets (either Se or placebo) daily with 12 hours interval, beginning from the first day of HDC through 14 days after HSCT. The HDC regimen included busulfan 4 mg/kg/d orally in divided doses for 4 days followed by cyclophosphamide 60 mg/kg intravenously once daily for 2 days. One day after completion of chemotherapy, patients received peripheral blood hematopoietic stem cell transplants from HLA-matched sibling donors.

Blood samples were collected three times during each patient’s hospital stay: before starting HDC (prior to first dose of Se), 7 days (+7) and 14 days (+14) after HSCT. Samples were collected in citrated tubes and centrifuged for 10 min within 2 hours of sampling. Plasma was removed and stored at -70˚C until assay. TNF-α, IL-1β, and IL-6 plasma levels were determined using high sensitivity ELISA kits (Bender Med, Vienna, Austria), following manufacturer’s instructions.

Values are expressed as mean ± SEM. Changes in plasma cytokines levels over time and as a function of group, were analyzed by performing the repeated measures of variance (ANOVA). *P-*value < 0.05 was considered as a significant difference.

Seventy-seven patients entered the study. Among these, 3 patients discontinued and 74 patients completed the study. Baseline characteristics of the patients were similar in both study groups [10]. In each group there were 37 patients (16 females and 21 males) with median age of 32 years (ranged 18–55).

Results of cytokines measurements in both study groups are summarized in Table 1. Due to some technical faults, some of the samples were not available and the number of analyzed samples varied for each cytokine. The mean plasma levels of TNF-α, IL-1β, and IL-6 were not significantly different between study groups at any sampling time point. The pattern of change in the cytokines levels was similar between two groups as well (there was no significant group-time interaction). TNF-α level declined slightly (*P* = 0.14) from baseline to day +7 and increased significantly within the next 7 days (*P* = 0.03) in the entire patient group. IL-1β level significantly decreased from baseline to day +7 (*P* < 0.001) and increased within the next 7 days (*P* = 0.89). Unlike the above cytokines, IL-6 level significantly increased from baseline to day +7 (*P* < 0.001) and dropped dramatically during the next 7 days (*P* < 0.001).

**Table 1:** Changes in plasma cytokines concentrations in study groups*

**Cytokine**	**Selenium group**	**Control group**	P-value**
**TNF-α**			
No. of patients	37	34	
Baseline conc. (pg/ml)	2.02 ± 0.09	2.00 ± 0.10	
+ 7 conc. (pg/ml)	1.98 ± 0.08	1.86 ± 0.08	0.13
+ 14 conc. (pg/ml)	2.18 ± 0.11	2.00 ± 0.11	
**IL-1β**			
No. of patients	32	28	
Baseline conc. (pg/ml)	0.28 ± 0.01	0.29 ± 0.02	
+ 7 conc. (pg/ml)	0.25 ± 0.01	0.25 ± 0.01	0.88
+ 14 conc. (pg/ml)	0.26 ± 0.01	0.26 ± 0.01	
**IL-6**			
No. of patients	34	29	
Baseline conc. (pg/ml)	3.43 ± 0.67	2.83 ± 0.55	
+ 7 conc. (pg/ml)	10.18 ± 0.67	9.77 ± 0.75	0.96
+ 14 conc. (pg/ml)	4.54 ± 0.72	4.73 ± 0.74	

*Data are reported as mean ± SEM.

**Comparison of each cytokine levels on day +7 and +14 between Se and control group.

Results of our current study confirmed the changes in pro-inflammatory cytokines levels after HSCT. The significant increase in IL-6 level within few days after transplantation, which was detected in all our study subjects, was also reported by Wang et al. [11] and Melenhorst et al. [12]. The slight decrease in TNF-α level during the first week of transplantation and the subsequent elevation in its level, were demonstrated in Min et al.’s study as well [13]. In addition, the pattern of change in IL-1β level was similar to TNF-α, a finding that is consistent with Melenhorst et al. [12] study.

On the other hand, despite a significant increase in serum Se concentration and Glu.Px activity in Se group, which we have reported previously [10], no significant differences in cytokines levels were observed between two groups at either time points. Two possible reasons could be suggested to justify our findings. According to our previous report, Se level and Glu.Px activity were not significantly different between study groups until the third week of Se supplementation. This implies that while ROS and pro-inflammatory cytokines were generating during HDC, Glu.Px activity in Se group was not significantly different with the control group. This finding probably explains why the improvement in anti-oxidative status of patients receiving Se did not alter the pro-inflammatory cytokines levels in comparison with the control group. Hence, earlier initiation or larger doses of Se might be required to detect the anti-inflammatory effects of Se. In addition, based on Fall-Dickson et al. study [14], local expression of pro-inflammatory cytokines might be a more accurate marker for evaluating anti-inflammatory effects of Se. In that study, TNF-α concentration in plasma, saliva, and buccal epithelial cells was measured at baseline and 9 days after conditioning chemotherapy in HSCT patients. Significant differences in the cytokine concentrations on day 9 were found between the three samples. Evaluating oral pain at the same time point, they suggested that the local level of TNF-α is a more reliable indicator of oral mucositis severity. Therefore, in our study, it is likely that Se supplementation resulted in alteration of cytokines levels in local tissue rather than peripheral blood.

Based on our results, inflammatory status of the patients in Se group did not improve; however they did benefit from Se supplementation. Fischer et al. [15] have defined a role for Se in the repair of the cells’ DNA after their exposure to radiation or chemotherapy drugs. Selenium exerts this function through a p53-dependent pathway which is absent in tumor cells due to lack of wild-type p53. They suggested this selective chemo-protection as a mechanism to alleviate toxicities induced by anti-cancer treatments especially DNA-damaging agents. Following administration of these agents, gut epithelium and bone marrow are highly susceptible to DNA damage due to their rapid proliferation. In our study all the subjects received HDC consisted of busulfan and cyclophosphamide, both of which are DNA-damaging chemotherapeutics. In agreement with the results of Fischer et al. [15] study, following administration of Se, significant reduction in the severity of mucositis as well as a marginally significant decrease in duration of neutropenia was observed in our study.

In summary, results of the current study suggests that Se supplementation can prevent severe OM in HSCT setting by mechanisms other than reducing production of pro-inflammatory cytokines; however samples from local tissues are also required to confirm our results. We also hypothesize that earlier administration and/or using larger doses of Se would result in optimum desired effects of Se supplementation in this setting. On the other hand, the observed results could be due to the small sample size of the study as well. Larger randomized controlled trials are recommended to evaluate these concepts.

## Abbreviations

TNF-α: Tumor necrosis factor alpha; IL-1β: Interleukin-1β; IL-6: Interleukin-6; HSCT: Hematopoietic stem cell transplantation; Se: Selenium; HDC: High dose chemotherapy; GVHD: Graft-versus-host disease; OM: Oral mucositis; Glu.Px: Glutathione peroxidase.

## Competing interests

The authors declare that they have no competing interests.

## Authors’ contributions

All authors contributed to developing the study protocol. In addition, ND gathered blood samples, analyzed some part of data and drafted the manuscript. MR contributed to interpretation of the results and revising the manuscript. ZJ contributed to the implementing the trial, analysis of blood samples and drafting the manuscript. MH supervised the whole project, contributed to interpretation of the results and revised the manuscript. AG supervised the whole project, contributed to the data interpretation. All authors have approved the final version of the manuscript.

## References

[B1] Hatzimichael E Tuthill M Hematopoietic stem cell transplantation Stem Cells Cloning: Adv Appl 2010 3 105 117 10.2147/SCCAA.S6815 PMC3781735 24198516

[B2] Tabbara IA Zimmerman K Morgan C Nahleh Z Allogeneic hematopoietic stem cell transplantation: complications and results Arch Intern Med 2002 162 1558 1566 10.1001/archinte.162.14.1558 12123398

[B3] Schots R Kaufman L Van Riet I Othman TB De Waele M Van Camp B Demanet C Proinflammatory cytokines and their role in the development of major transplant-related complications in the early phase after allogeneic bone marrow transplantation Leukemia 2003 17 1150 1156 10.1038/sj.leu.2402946 12764383

[B4] Logan RM Stringer AM Bowen JM Yeoh AS-J Gibson RJ Sonis ST Keefe DM The role of pro-inflammatory cytokines in cancer treatment-induced alimentary tract mucositis: pathobiology, animal models and cytotoxic drugs Cancer Treat Rev 2007 33 448 460 10.1016/j.ctrv.2007.03.001 17507164

[B5] Takatsuka H Takemoto Y Yamada S Wada H Tamura S Fujimori Y Okamoto T Suehiro A Kanamaru A Kakishita E Complications after bone marrow transplantation are manifestations of systemic inflammatory response syndrome Bone Marrow Transplant 2000 26 419 426 10.1038/sj.bmt.1702517 10982289

[B6] Blackwell TS Christman JW Hagan T Price P Edens T Morris PE Wolff SN Goodman SA Christman BW Oxidative stress and NF-κ B activation: correlation in patients following allogeneic bone marrow transplantation Antiox Redox Signal 2000 2 93 102 10.1089/ars.2000.2.1-93 11232605

[B7] Spencer A Horvath N Gibson J Prince H Herrmann R Bashford J Joske D Grigg A McKendrick J Prosser I Prospective randomised trial of amifostine cytoprotection in myeloma patients undergoing high-dose melphalan conditioned autologous stem cell transplantation Bone Marrow Transplant 2005 35 971 977 10.1038/sj.bmt.1704946 15778725

[B8] Levine JE Paczesny S Mineishi S Braun T Choi SW Hutchinson RJ Jones D Khaled Y Kitko CL Bickley D Etanercept plus methylprednisolone as initial therapy for acute graft-versus-host disease Blood 2008 111 2470 2475 10.1182/blood-2007-09-112987 18042798 PMC2361693

[B9] Duntas L Selenium and inflammation: underlying anti-inflammatory mechanisms Horm Metab Res 2009 41 443 447 10.1055/s-0029-1220724 19418416

[B10] Jahangard-Rafsanjani Z Gholami K Hadjibabaie M Shamshiri A Alimoghadam K Sarayani A Mojtahedzadeh M Ostadali-Dehaghi M Ghavamzadeh A The efficacy of selenium in prevention of oral mucositis in patients undergoing hematopoietic SCT: a randomized clinical trial Bone Marrow Transplant 2013 48 832 836 10.1038/bmt.2012.250 23292233

[B11] Wang XS Shi Q Williams LA Cleeland CS Mobley GM Reuben JM Lee BN Giralt SA Serum interleukin-6 predicts the development of multiple symptoms at nadir of allogeneic hematopoietic stem cell transplantation Cancer 2008 113 2102 2109 10.1002/cncr.23820 18792065 PMC2633777

[B12] Melenhorst JJ Tian X Xu D Sandler NG Scheinberg P Biancotto A Scheinberg P McCoy JP Hensel NF McIver Z Cytopenia and leukocyte recovery shape cytokine fluctuations after myeloablative allogeneic hematopoietic stem cell transplantation Haematologica 2012 97 867 873 10.3324/haematol.2011.053363 22133778 PMC3366652

[B13] Min C Lee W Min D Lee D Kim Y Park Y Kim H Lee S Kim D Lee J The kinetics of circulating cytokines including IL-6, TNF-alpha, IL-8 and IL-10 following allogeneic hematopoietic stem cell transplantation Bone Marrow Transplant 2001 28 935 940 10.1038/sj.bmt.1703258 11753547

[B14] FalI-Dickson JM Ramsay ES Castro K Woltz P Sportès C Oral mucositis-related oropharyngeal pain and correlative tumor necrosis factor-α expression in adult oncology patients undergoing hematopoietic stem cell transplantation Clin Ther 2007 29 2547 2561 10.1016/j.clinthera.2007.12.004 18164921

[B15] Fischer JL Mihelc EM Pollok KE Smith ML Chemotherapeutic selectivity conferred by selenium: a role for p53-dependent DNA repair Mol Cancer Ther 2007 6 355 361 10.1158/1535-7163.MCT-06-0472 17237294

